# Acupuncture and Moxibustion have Different Effects on Fatigue by Regulating the Autonomic Nervous System: A Pilot Controlled Clinical Trial

**DOI:** 10.1038/srep37846

**Published:** 2016-11-25

**Authors:** Qing Shu, Hua Wang, Daniela Litscher, Song Wu, Li Chen, Ingrid Gaischek, Lu Wang, Wenjuan He, Huanjiao Zhou, Gerhard Litscher, Fengxia Liang

**Affiliations:** 1 Acupuncture & Moxibustion Institute, Hubei University of Chinese Medicine, Wuhan (430061), China; 2 Hubei Provincial Collaborative Innovation Center of Preventive Treatment by Acupuncture & Moxibustion, Wuhan (430061), China; 3 TCM Research Center Graz, Research Unit for Complementary and Integrative Laser Medicine, Research Unit of Biomedical Engineering in Anesthesia and Intensive Care Medicine, Medical University of Graz, Graz (8036), Austria

## Abstract

In order to investigate the different effects of acupuncture and moxibustion on chronic fatigue syndrome (CFS) and alterations in the autonomic nervous system by measuring heart rate variability (HRV). Forty-five participants were recruited and randomly divided into 3 groups using a randomization schedule. The control group (CG, n = 15) and the acupuncture group (AG, n = 15) were treated by manipulation acupuncture, and the moxibustion group (MG, n = 15) was treated by indirect moxibustion. Primary outcomes were the scores of the Fatigue Assessment Instrument (FAI). Secondary outcomes were the HRV parameters which can reflect activity of the autonomic nervous system. This trial considered both instantaneous changes and long-term effectiveness. FAI scores decreased after the 4th and 10th treatments in the 3 groups. The decrease in FAI in the MG was greater than that in the AG. Acupuncture was more effective in instantaneous changes of HRV and moxibustion in long-term aspects. Both acupuncture and moxibustion improved fatigue in CFS patients, but moxibustion was more effective. The possible mechanism of the intervention may be through activation of the vagus nerve. Moxibustion was more effective than acupuncture in long-term treatment of CFS.

Chronic fatigue syndrome (CFS), which is also known as myalgic encephalomyelitis, is a clinically defined condition characterized by severe disabling fatigue and a combination of symptoms that prominently feature self-reported impairments in concentration and short-term memory, sleep disturbances, and musculoskeletal pain^1^. CFS is thought to be prevalent worldwide, with incidence varying from 0.1 to 1%, and more females than males are affected^2^,^3^,^4^. It poses considerable challenges to both patients and those close to them including health care providers and society in general. CFS markedly lowers the quality of life of affected patients. Until now, no single cause of CFS has been discovered. However, various debatable hypotheses have been considered, including pathological^5^, immunological^6^, psychological^7^, and neuroendocrine^8^ factors. Based on evidence from randomized control trials (RCTs), different types of treatments such as graded exercise therapy and cognitive behavior therapy have been found to reduce symptoms and improve function in CFS patients. Other interventions including pharmacological treatments, supplements, as well as complementary and alternative therapies have demonstrated limited evidence of effectiveness^9^,^10^,^11^. Studies of traditional Chinese medicine (TCM), which includes Chinese herbal medicine, acupuncture, moxibustion, qigong, and acupoint application, have shown that TCM alone or in combination with other interventions can significantly alleviate fatigue symptoms^12^. Acupuncture is one of the most popular complementary and alternative therapies in Western countries, and its application is increasing over time. Moxibustion is also an oriental treatment that is often combined with acupuncture, which effectively invigorates health in TCM theories. It has no effect on blood chemistry or urine and is safe for clinical use^13^. Acupuncture has been widely applied in CFS, and outcomes have shown its effectiveness. However, the qualities of these studies were generally poor and few used the RCT design^14^. Only a few studies were included in systematic reviews of complementary and alternative medicine for patients with CFS^15^,^16^.

The autonomic nervous system (ANS) is an important regulator of many physiological functions^17^. Many studies have demonstrated an impairment of the ANS in patients with CFS^18^. The ANS can be investigated noninvasively by recording heart rate (HR) and then calculating heart rate variability (HRV) with Fourier analysis^19^. This method has been used in investigating CFS patients and has revealed differences between CFS patients and healthy people^20^. Acupuncture has been shown to influence the central nervous system^21^ and the functional status of the ANS in humans^22^,^23^ and laboratory animals^24^. Acupuncture has also been shown to be effective in modulating HRV, although there was not sufficient evidence to determine if HRV could be an indicator of the therapeutic effect of acupuncture^25^.

In light of advances in research methods and the shortcomings of previous clinical trials studying acupuncture treatment of CFS and regulation of HRV, we conducted a pilot controlled clinical trial. The aim of the trial was to investigate different effects of acupuncture and moxibustion on fatigue and regulation of HRV in patients with CFS. In addition, we prepared for the next joint RCT between researchers in Graz, Austria and Wuhan, China.

## Methods

This was a controlled clinical trial, which was conducted at Hubei University of Chinese Medicine and the TCM Research Center Graz, Medical University of Graz. The study protocol was approved by the Medical Ethics Committee of Hubei University of Chinese Medicine on July 5, 2014. The Chinese Clinical Trial Registry (www.chictr.org.cn) which is a publically accessible primary register that participates in the WHO International Clinical Trial Registry Platform checked our trial after we submitted all research data to www.medresman.org and retrospectively registered with No. ChiCTR-IPR-15007383 on November 12, 2015. The authors confirm that all ongoing and related trials for this intervention are registered. All methods were performed in accordance with CONSORT, STRICTA, and STRICTOM guidelines^26^,^27^,^28^. Supporting checklists are available as supplementary materials. Participants provided written informed consent.

From October 1, 2014 to May 31, 2015, we used recruitment notices to recruit 45 participants including healthy volunteers and patients with CFS from Huangjiahu Hospital, which is affiliated with Hubei University of Chinese Medicine, and the External Treatment Center, which is affiliated with the Hubei Provincial Collaborative Innovation Center of Preventive Treatment by Acupuncture and Moxibustion. The trial included patients with CFS that were 18 to 65 years old, who met the diagnostic criteria for CFS that were published by the Centers for Disease Control and Prevention in the United States^1^ and had no skin injuries at the acupoints ST36 (Zusanli), CV4 (Guanyuan). Exclusion criteria are shown in Table 1. Healthy volunteers were recruited to compare the effects of acupuncture between patients with CFS and a healthy control group.

### Randomization

A randomization schedule was provided by SPSS (version 20.0), and the randomized numbers were sealed in an envelope. After signing the informed consent, patients with CFS received the sealed envelope and were randomly assigned to the acupuncture group (AG) or moxibustion group (MG) according to the random number. The healthy volunteers in the control group (CG) only signed the informed consent. Baseline characteristics of the participants are shown in Table 2.

### Interventions

**Figure 1:**
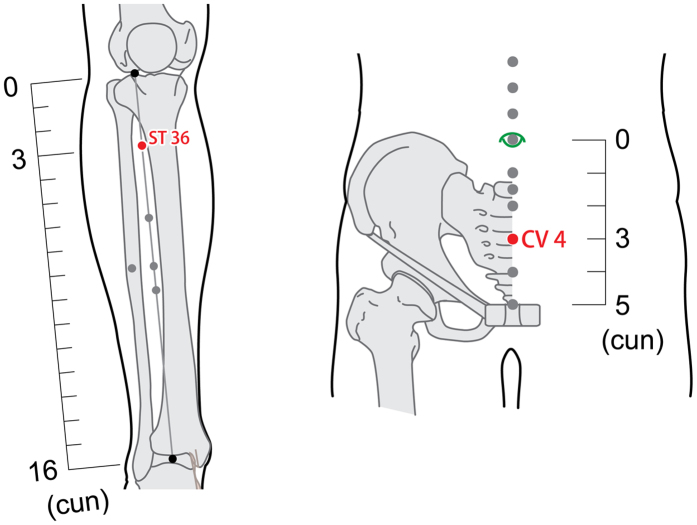
Acupoint locations.

**Figure 2:**
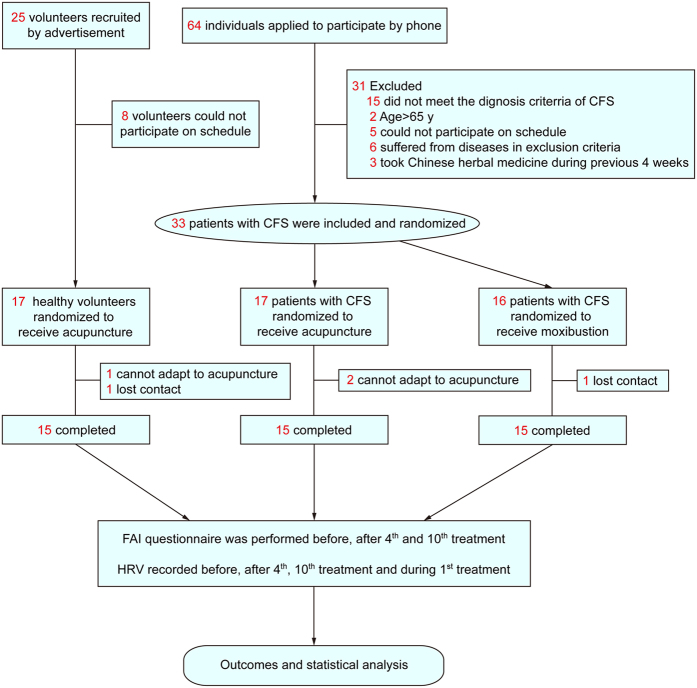
Flow of participants.

We use the TCM style of acupuncture and moxibustion. Two acupuncturists, who had at least 10 years of acupuncture and moxibustion experience, participated in the trial. Both of the acupuncturists were members of the College of Acupuncture and Moxibustion, Hubei University of Chinese Medicine, and were registered as medical practitioner acupuncturists by the National Health and Family Planning Commission of China. We selected acupoints ST36 (bilateral) and CV4 (Fig. 1), which can reinforce qi in TCM theories and ameliorate fatigue in Western medical systems^29^,^30^. In the CG and AG, 3 acupoints were stimulated by single-use stainless steel needles (0.30 mm × 40 mm, Global, Su Zhou Acupuncture Goods Co., Ltd, Suzhou, China). Each intervention session lasted 15 minutes, and the sessions were performed identically in both groups. The acupuncturist manipulated the needle by lifting-thrusting and twirling-rotating every 5 minutes to maintain the Deqi sensation, which means that the participants felt soreness, aching, and deep pressure as described in the Massachusetts General Hospital acupuncture sensation scales (MASS)^31^. The moxibustion group received warming from indirect moxibustion using a commercially available moxibustion box. The box had a cylindrical opening to hold a pillar of moxa stick and an elastic cord to affix it to the acupoint. The moxa stick (Φ18 × 200 mm) was wrapped with moxa floss, which is white, soft, cotton-like fibers prepared from moxa leaves. During the treatment, participants were asked to roll up their pants and lie on a bed in a supine position. Moxa boxes containing a pillar of moxa stick were affixed to the acupoints. The moxa was burned about 1 to 2 cm above the acupoint skin for 15 minutes, which can make the patient feel warm continuously. Burning injury was carefully avoided in the process by the acupuncturist focusing his attention and whisking away the burning ash in a timely manner. Every participant in the 3 groups received 10 sessions, which were administered every other day (Fig. 2).

### Outcome measurements

Primary outcomes were the scores of the Fatigue Assessment Instrument (FAI) recommended by Joseph E. Schwartz in 1993^32^, which can appropriately reflect the level of fatigue^33^. The FAI is a 29-item rating scale consisting of 4 underlying constructs: the severity of fatigue, the sensitivity of one’s fatigue in a particular circumstance (such as heat, cold, or stress), the consequences of fatigue, and the response of fatigue to rest and/or sleep. The response key for each item was a 7-point scale ranging from “1 = completely disagree” to “7 = completely agree;” the total score of each item was the level of fatigue. This scale included not only the severity of fatigue, but the influence of fatigue on quality of life as well. The questionnaire was completed before and after the 4^th^ and 10^th^ treatments.

**Figure 3:**
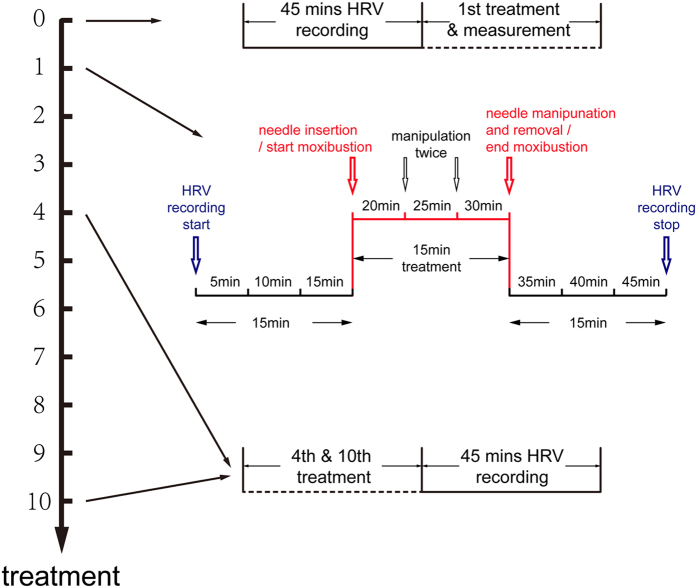
Procedure of Treatment & Measurement.

Secondary outcomes were the HRV parameters including mean heart rate (HR), standard deviation of normal sinus beat (NN) intervals (SDNN), total HRV, low-frequency (LF), high-frequency (HF), and lg (LF/HF). HRV was measured by a portable device (Medilog AR12 HRV, Huntleigh Healthcare, Cardiff, UK) from the TCM Research Center at the Medical University in Graz. Prior to the start of treatment or HRV recording, room temperature was adjusted to keep the participant comfortable (24–26 °C). Noises and strong light were excluded. In other words, the surroundings were kept stable state. Participants lay on a comfortable bed for 15–20 min to allow their HR and breathing to stabilize. All the participants were informed not to take food or drinks containing caffeine before or during the study, such as coffee or tea, which have the function of exciting the nervous system. The device used 3 adhesive electrodes that were applied to the chest. The duration of normal sinus beat RR-intervals was measured during 5 minutes, and spectral analysis of HRV was performed and stored on a memory card. After removing the card from the portable device, HRV data were read by a card reader and transferred to the center in Graz, Austria via the internet. HRV data were then analyzed and a PDF report was sent back to Wuhan, China. To assess long-term effectiveness, HRV was analyzed for 45 minutes before treatment, and after the 4^th^ and 10^th^ treatments. Instantaneous changes in HRV were assessed 3 times for 15 minutes during the 1^st^ treatment, which included determining HRV parameters before, during, and after treatment (Fig. 3).

### Statistical analysis

Analysis was performed by blinded biostatisticians using SPSS software. The correlation between FAI scores and HRV parameters was analyzed by Pearson Correlation Coefficient (PCC). FAI scores assessed before and after the 4^th^ and 10^th^ treatments were analyzed by repeated measures ANOVA to reflect the improvement in fatigue syndrome by acupuncture or moxibustion. Differences among the 3 groups were analyzed by one-way ANOVA. To assess the difference between 2 groups, post-hoc multiple comparisons were conducted if there were differences in the 3 groups. The HRV parameters recorded during the 1^st^ treatment were analyzed by repeated measures ANOVA and post-hoc multiple comparisons to assess instantaneous changes in HRV. The long-term effectiveness of HRV parameters was analyzed by repeated measures ANOVA and post-hoc multiple comparisons using parameters obtained before and after the 4^th^ and 10^th^ treatments. Post-hoc power analyses were conducted after ANOVA to observe the power. *P* values less than 0.05 were considered statistically significant and tests were two-sided.

## Results

In all, 64 CFS patients and 25 healthy volunteers were willing to participate in the trial. Thirty-one CFS patients (48.4%) were ineligible according to the inclusion and exclusion criteria; 8 healthy volunteers (32%) could not participate on schedule. Thus, 50 participants were recruited to participate in this trial. Three CFS patients and 2 healthy volunteers were lost during the treatment because they could not adapt to acupuncture or they lost contact. Forty-five participants completed the trial on schedule, and each group had 15 participants (Fig. 2). There were no significant differences in age and sex ratio between the 3 groups, and no significant differences in FAI scores were observed between AG and MG, which were similar at baseline (Table 2).

### HRV parameters had a weak correlation with the level of fatigue

**Figure 4:**
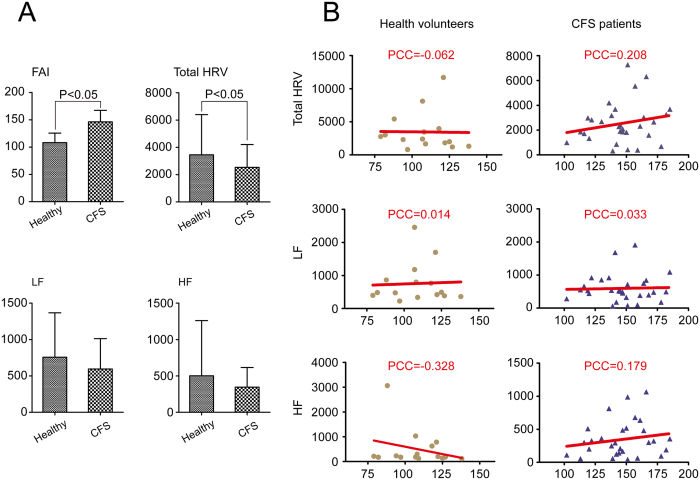
correlation between FAI scores and HRV parameters. (**A**) The difference between healthy volunteers and CFS patients in FAI and HRV indices. Compared with the healthy volunteers, the quantitative value of total HRV was significantly lower and FAI scores was significantly higher in CFS patients (*P* < 0.05). (**B**) The correlation coefficient between HRV indices and FAI scores. All the HRV indices had a weak correlation with the level of fatigue either in the CFS patients or health volunteers. Total HRV and HF in CFS patients as well as HF in health volunteers showed a closer correlation than other parameters. (PCC: Pearson correlation coefficient, X axis in Figure (4b) represent the FAI scores).

The correlation between FAI scores and HRV parameters is presented in Fig. 4, which shows that all the HRV indices had a weak correlation with the level of fatigue, in the CFS patients as well as in the healthy volunteers. In particular, total HRV and HF in CFS patients as well as HF in healthy volunteers showed a higher correlation than other parameters. Compared with the healthy volunteers, the quantitative value of total HRV was significantly lower and FAI scores were significantly higher in CFS patients (*P* < 0.05). There was no significant difference in LF and HF between healthy volunteers and CFS patients.

PCC: Pearson correlation coefficient, X axis in Fig. (4b) represent the FAI scores.

### Acupuncture and moxibustion improved fatigue, while moxibustion was more effective after the 10th treatment

**Figure 5:**
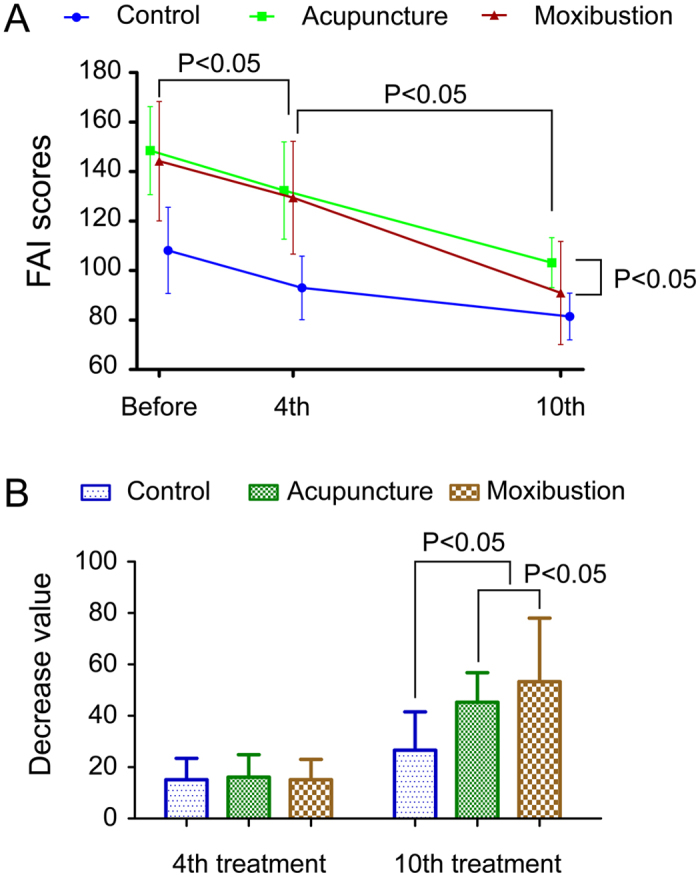
Changes in FAI. (**A**) FAI scores before and after 4^th^ and 10^th^ treatments. FAI decreased after the 4^th^ and 10^th^ treatments in the 3 groups. FAI value at the 10^th^ treatment was lower than that at the 4^th^ (*P* < 0.05). (**B**) The decrease in FAI value of 3 groups. There were no significant differences in decrease value after 4^th^ treatment (*P* > 0.05). After the 10^th^ treatment, decrease value in MG were more pronounced than that in AG, while decrease value in AG were more pronounced than that in CG (*P* < 0.05).

The FAI scores of the participants before and after the 4^th^ and 10^th^ treatments and the decrease in values after the 4^th^ and 10^th^ treatments are shown in Fig. 5. The FAI scores of AG, MG, and CG decreased significantly after the 4^th^ and 10^th^ treatments. In particular, the FAI score in MG was lower than that in AG after the 10^th^ treatment (Fig. 5a). Compared with the healthy volunteers, the values of the decreased scores were higher in the CFS patients after the 10^th^ treatment, while the decrease values in MG were higher than that in AG (Fig. 5b).

### Acupuncture could change HRV parameters more effectively than moxibustion during the 1st treatment

**Figure 6:**
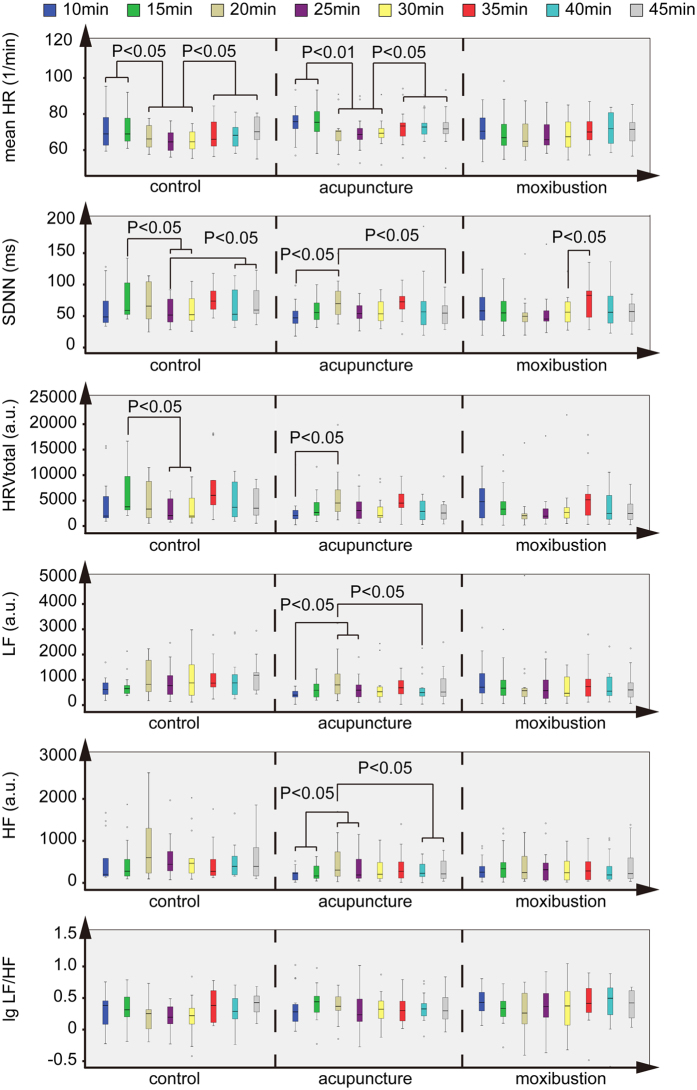
Instantaneous Changes of HRV parameters during the 1st treatment. During the 1^st^ treatment, the mean HR in CG and AG decreased after the needle was inserted and increased after the needle was removed, and it did not change in MG. SDNN in CG and AG increased after the needle was inserted and decreased after the needle was removed, and it only increased between 30 minutes and 35 minutes in MG. Total HRV decreased in CG and increased in AG after the needle was inserted, and it did not change in MG. LF and HF both increased after the needle was inserted and decreased after the needle was removed in AG, and did not change in MG. lg LF/HF did not show regular changes during the ^st^ treatment.

The instantaneous changes in HRV caused by acupuncture and moxibustion during the 1^st^ treatment are shown in Fig. 6. Because of instability in the parameters during the first 5 minutes, which were influenced by activity before treatment, the first 5 minutes were excluded from analysis of instantaneous change. Mean HR decreased significantly after acupuncture in CG and AG, and did not change after moxibustion in MG. SDNN decreased significantly after the needle was inserted and increased after the needle was removed in CG and AG, and only increased after moxibustion ended in MG. Total HRV increased significantly after the needle was inserted in AG and decreased in CG. LF increased significantly (P < 0.05) after the needle was inserted (20 min period) in AG, and did not change significantly in CG and MG. The HF of AG increased significantly after the needle was inserted and decreased after the needle was removed. In contrast, the HF of CG and MG did not change significantly during the 1^st^ treatment. lg LF/HF did not show regular changes during the 1^st^ treatment.

### Moxibustion could change HRV parameters more effectively than acupuncture in the long-term

**Figure 7:**
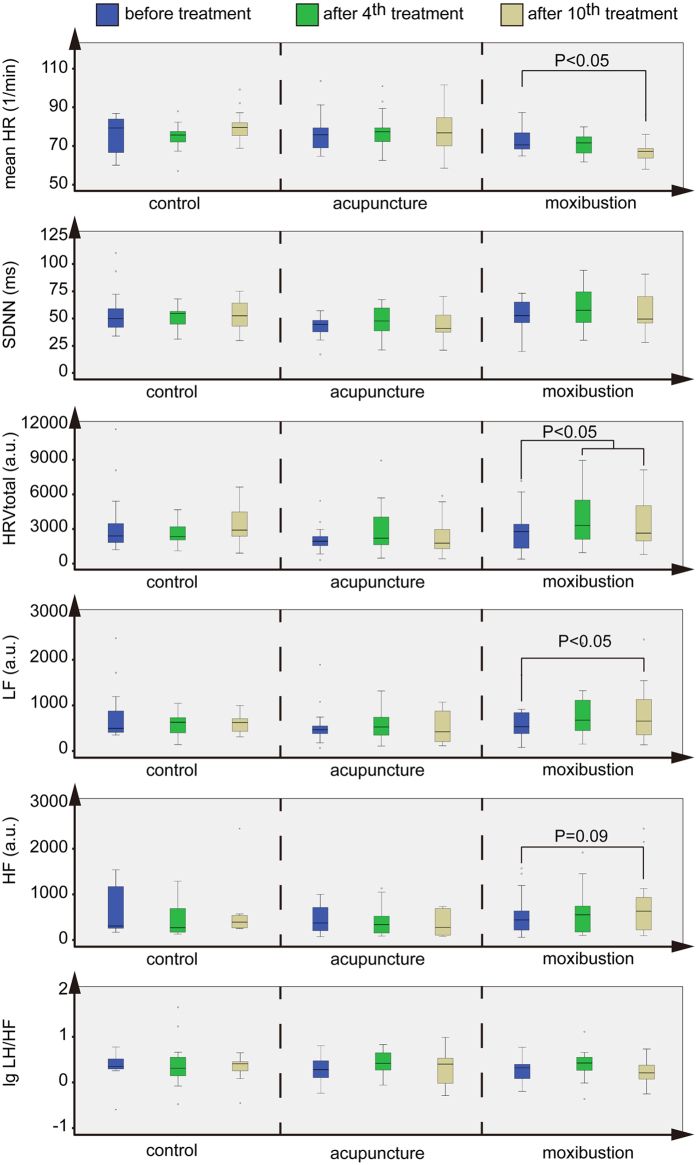
Long-term effectiveness of HRV parameters before and after the 4th and 10th treatments. Mean HR decreased after the 10^th^ treatment in MG. Total HRV and LF increased significantly after the 4^th^ or 10^th^ treatment in MG (P < 0.05). The increase in HF in MG was not significant (P = 0.09). There was no significant effectiveness in CG and AG.

The long-term effects of acupuncture and moxibustion on HRV are summarized in Fig. 7. Mean HR did not change significantly in CG and AG, but decreased in MG after 10 treatments. The SDNN did not change significantly in the 3 groups. Compared with the 1^st^ treatment, the total HRV increased significantly after the 4^th^ and 10^th^ treatments in MG, and it did not change in CG and AG. Compared with the 1^st^ treatment, the LF of the 10^th^ treatment increased significantly in MG, and it did not change in CG and AG. The HF did not change in the 3 groups, but the tendency to increase between the 1^st^ and 10^th^ treatments emerged in MG; however, this effect was not significant (P = 0.09). The lg LF/HF did not change in the 3 groups.

## Discussion

A systematic review of acupuncture and moxibustion treatment for CFS in China argued that acupuncture and moxibustion were effective in treating CFS, but the conclusion was based on poor quality studies and was not supported by meta-analysis^14^. Our outcomes were based on a study design that was randomized and controlled, and the acupuncturists and biostatisticians were separated to avoid bias. In this trial, the acupuncturists and patients with CFS could not be blinded because of the characteristics of acupuncture and moxibustion. Therefore, we designed a strict procedure and required the acupuncturist to perform it precisely. In addition, participants who did not receive treatment on schedule were excluded. Although this was a pilot trial, we included elements of a RCT.

Although the correlations between FAI scores and HRV parameters were weak, the tendency that people with fatigue symptoms had lower HRV was present partially. The results of FAI scores showed that acupuncture could improve fatigue level in participants with or without CFS and that moxibustion was more effective. Despite the observation that healthy volunteers derived benefits from acupuncture, which could reduce their FAI scores, CFS patients showed greater improvement in fatigue. A tendency for FAI scores to decrease after the 4^th^ and 10^th^ treatments emerged in the 3 groups. This outcome showed that acupuncture and moxibustion could be effective for a short time, but the different levels of effectiveness of acupuncture and moxibustion did not emerge after a short time. The decrease in value at the 10^th^ treatment was greater than that at the 4^th^ treatment, which showed that acupuncture and moxibustion were more effective in the long-term than in the short-term. The FAI scores of MG were lower than those of AG after the 10^th^ treatment, which suggests that CFS patients could obtain more benefits from moxibustion than from acupuncture.

The ANS has 2 branches, the sympathetic nervous system and the parasympathetic nervous system. The sympathetic nervous system is responsible for flight and stress situations, while the parasympathetic nervous system is dominant when a person is relaxed e.g. when sleep^34^. As an invisible measurement, HRV, which can be assessed by spectral analysis in the time and frequency domains, can reflect beat-to-beat changes in HR. SDNN, which is the standard deviation of all NN intervals, primarily reflects circadian rhythms^19^. HF reflects activity of the parasympathetic nervous system, while LF is modulated by both the sympathetic and parasympathetic nervous systems^35^. The LF/HF ratio can assess the state of the sympatho-vagal balance modulating the sino-atrial node under numerous types of physiological and physiopathological conditions^36^. Total HRV is the summary of the frequency domain indices including HF, LF, very-low-frequency (VLF), and ultra-low-frequency (ULF).

CFS patients always exhibit impairments in the ANS. A study showed that CFS patients had a significantly higher mean HR, reduced LF, VLF, and total HRV, which suggests a state of sympathetic ANS predominance^37^. Although the current study did not show this type of results, the tendency showed a parallel to this previous work. Acupuncture modulation of HRV has been proven by a meta-analysis, which suggested that the LF but not the HF component could be modulated^25^. Thus, acupuncture can modulate the ANS by activating the sympathetic and parasympathetic nervous systems. However, differences between short-term and long-term effects as well as between acupuncture and moxibustion were not considered. In our trial, the parameters of HRV in different sessions modulated by acupuncture and moxibustion were analyzed to investigate instantaneous and long-term effectiveness.

In observing instantaneous changes, we found that the mean HR of patients with CFS decreased, while LF, HF, and total power (TP) increased to a certain extent after acupuncture. In contrast, the patients treated by moxibustion did not show significant changes. The LF/HF ratio showed polarized and irregular changes, from which we cannot draw a conclusion. The instantaneous changes of HRV showed that acupuncture was more effective than moxibustion in modulating the ANS, especially in activating the vagus nerve, which contains parasympathetic nervous fibers, and has the function of reducing HR.

The long-term effectiveness showed another trend. These results showed that moxibustion could also activate the vagus nerve, which was reflected by LF for both continuous and cumulative effects. These types of changes may be responsible for the alterations in FAI scores, which showed that moxibustion could improve fatigue better than acupuncture in the long-term. Although the HF did not show significant changes in the 3 groups, the decreased value of HF in MG was higher than that in CG and AG and the *P* value was close to 0.05. Given that the sample size was 15 in each group, the changes may become significant after increasing the sample size. The tendency for HF to increase may be responsible for improvement in fatigue, which means that activating the parasympathetic nervous system could also be achieved by moxibustion in improving fatigue.

The instantaneous change and long-term effectiveness of different interventions showed disparate outcomes in HRV indices. These differences may be attributed to the characteristics of acupuncture and moxibustion. In TCM theories, the acupuncture needle exerts stronger stimulation and quicker effectiveness and has the features of eliminating pathogenic factors and being instantaneous. In contrast, moxibustion is a warm, moderate, and durative stimulation and has the features of being reinforcing and continuous. Temperature at the surface of the skin was still higher after extinguishing moxibustion than it had been before treatment^38^. These may explain the disparate outcomes between instantaneous change and long-term effectiveness.

In TCM theory, ST36 and CV4 are regarded as reinforcing acupoints. ST36 can improve fatigue both in experimental animals^39^ and exercise-induced fatigue^29^,^40^. One possible mechanism underlying recovery from fatigue may involve increasing carnitine and glutathione in muscle^41^. Indirect moxibustion on CV4 has been shown to improve chronic fatigue, which suggests that modification of antioxidant activity may be one possible mechanism underlying the anti-fatigue effect^30^.

As this was a pilot trial, the sample size we calculated using the Power Analysis and Sample Size software package (PASS version 11.0). In the future large-size RCT protocol, sample size will be calculated according to rigorous methodology and considered in the improvement rate^42^. Because of ethical principles and actual clinical conditions in China, neither a control group of individuals who did not receive treatment nor a placebo group who received sham acupuncture and moxibustion were included. Nevertheless, the STRICTA and STRICTOM guidelines were followed strictly through the trial, and the outcomes still showed meaningful and effective treatment corresponding to other RCTs^25^. In addition, the instantaneous changes and long-term effectiveness may reflect the different mechanisms and characteristics of intervention of acupuncture and moxibustion in the treatment of CFS. However, HR is not only modulated by ANS but also by higher centers, such as the dorsal nucleus of vagus nerve and nucleus ambiguous in medulla oblongata. The application of fMRI which can physiologically monitor the peripheral response on time may be performed to explore the reaction of the ANS function^43^. Questionnaires about mood scales or mindfulness or direct EEG recordings might produce more specific parameters of the ANS. Combined with these enhanced detective methods, the positive results from this study should lay a foundation for the next joint study between researchers in Graz, Austria and Wuhan, China.

## Conclusion

The HRV indices which represent the activities of the ANS can reflect the fatigue to an extent, but it cannot be used to gauge the level of fatigue. Both acupuncture and moxibustion can improve fatigue of CFS patients. The mechanism responsible for this effect may involve activating the vagus nerve. Modulation of the sympathetic nervous system may be involved in moxibustion treatment of CFS as well. Moxibustion is more effective than acupuncture in treating CFS in the long-term, and this advantage is reflected by HRV indices.

## Additional Information

**How to cite this article**: Shu, Q. *et al*. Acupuncture and Moxibustion have Different Effects on Fatigue by Regulating the Autonomic Nervous System: A Pilot Controlled Clinical Trial. *Sci. Rep.* **6**, 37846; doi: 10.1038/srep37846 (2016).

**Publisher's note:** Springer Nature remains neutral with regard to jurisdictional claims in published maps and institutional affiliations.

## Figures and Tables

**Table 1:** Exclusion criteria.

A. Participants who could not accept or were afraid of acupuncture in the acupuncture group.
B. Participants who suffered from heart and cerebrovascular diseases, serious lung disease, diabetes, neurological diseases, or severe primary diseases such as those of the liver, kidney, and the hematopoietic system.
C. Participants who suffered from a mental health disorder, Alzheimer’s disease, or cancer.
D. Participants who had taken Chinese herbal medicine during the previous 4 weeks and preceding the investigation.
E. Pregnant women and nursing mothers.
F. Participants who suffered from thrombocytopenia and abnormalities of blood coagulation.
G. Participants with a history of alcohol or drug abuse.

**Table 2:** Baseline characteristics of participants in the trial.

Variable	Control (n = 15)	Acupuncture (n = 15)	Moxibustion (n = 15)
Age, mean (SD), y	37.73 (14.78)	38.07 (14.79)	37.33 (14.17)
Female, No. (%)	10 (66%)	11 (73%)	12 (80%)
Quantitative assessment of FAI, mean (SD)	108.1 (19.84)	148.5 (17.8)	144.2 (24.1)

SD … standard deviation; FAI … Fatigue Assessment Instrument.
